# Urinary TIMP-2 and IGFBP7 for the prediction of acute kidney injury following cardiac surgery

**DOI:** 10.1186/s12882-017-0592-8

**Published:** 2017-05-30

**Authors:** Yimei Wang, Zhouping Zou, Jifu Jin, Jie Teng, Jiarui Xu, Bo Shen, Wuhua Jiang, Yamin Zhuang, Lan Liu, Zhe Luo, Chunsheng Wang, Xiaoqiang Ding

**Affiliations:** 1 Departments of Nephrology, Zhongshan Hospital, Shanghai Medical College, Fudan University, No 180 Fenglin Rd, Shanghai, China; 2 Department of Cardiovascular Surgery, Zhongshan Hospital, Shanghai Medical College, Fudan University, No 180 Fenglin Rd, Shanghai, China; 3 Department of critical care medicine, Fudan University, No 180 Fenglin Rd, Shanghai, China

**Keywords:** Acute kidney injury, TIMP-2, IGFBP7, Cardiac surgery, Biomarker

## Abstract

**Background:**

Acute kidney injury (AKI) following cardiac surgery is common and associated with poor patient outcomes. Early risk assessment for development of AKI remains a challenge. The combination of urinary tissue inhibitor of metalloproteinase 2 (TIMP-2) and insulin-like growth factor binding protein 7 (IGFBP7) has been shown to be an excellent predictor of AKI following cardiac surgery, but reported studies are for predominately non-Asian populations.

**Methods:**

Adult patients were prospectively enrolled at Zhongshan hospital in Shanghai, China. The primary analysis was prediction of AKI and stage 2–3 AKI by [TIMP-2]*[IGFBP7] measured 4 h after postoperative ICU admission assessed using receiver operating characteristic curve (ROC) analysis. Kinetics of [TIMP-2]*[IGFBP7] following ICU admission were also examined.

**Results:**

We prospectively enrolled 57 cardiac surgery patients, of which 20 (35%) developed AKI and 6 (11%) developed stage 2–3 AKI. The area under the ROC curve (AUC) of [TIMP-2]*[IGFBP7] at 4 h after ICU admission was 0.80 (95% confidence interval (CI): 0.68–0.91) for development of AKI and 0.83 (95% CI: 0.69–0.96) for development of stage 2–3 AKI. Urinary [TIMP-2]*[IGFBP7] values at 4 h after ICU admission were significantly (*P* < 0.001) higher in patients who developed AKI than in patients who did not develop AKI (mean (standard error) of 1.08 (0.34) (ng/mL)^2^/1000 and 0.29 (0.05) (ng/mL)^2^/1000, respectively). The time-profile of [TIMP-2]*[IGFBP7] suggests the markers started to elevate by the time of ICU admission in patients who developed AKI and either decreased or remained flat in patients without AKI.

**Conclusion:**

The combination of urinary TIMP-2 and IGFBP7 4 h after postoperative ICU admission identifies patients at risk for developing AKI, not just stage 2–3 AKI following cardiac surgery.

## Background

Acute kidney injury (AKI) is a global health concern associated with increased morbidity and mortality [1–3]. Patients undergoing cardiac surgery, especially with cardiopulmonary bypass, are at high risk for AKI. A recent meta-analysis estimated the global incidence of AKI following cardiac surgery to be approximately 22% [4]. Development of AKI following cardiac surgery increases hospital and ICU length of stage, short and long term mortality, and risk of long-term renal dysfunction [4–6].

Despite the adverse consequences associated with AKI, missed or delayed diagnosis of AKI is common [7, 8]. Early diagnosis of AKI requires close monitoring of changes in serum creatinine and urine output, which may not be practical for all patients. Additionally, changes in these markers of renal function often lag injury [9]. Risk assessment for AKI is recommended by clinical practice guidelines to facilitate early detection of AKI and potentially prevent AKI in some cases [10]. Yet, few tools for early risk assessment are available beyond clinical evaluation.

Recently, a biomarker-based test for AKI risk assessment has become available for clinical use in Europe and the United States [11]. The test measures two urinary proteins involved in cell-cycle arrest: tissue inhibitor of metalloproteinase 2 (TIMP-2) and insulin-like growth factor-binding protein 7 (IGFBP7). These urinary cell cycle arrest markers are believed to be indicators of renal tubule cell stress involved in the earliest phases of acute kidney injury [12]. The combination of these biomarkers ([TIMP-2]•[IGFBP7]) has been shown to be predictive of AKI in large diverse cohorts of critically ill patients in the Sapphire, Opal and Topaz studies [12–14].

Studies in Germany have investigated the performance of urinary [TIMP-2]•[IGFBP7] in cardiac surgery patients [15–18]. Post-operative urinary [TIMP-2]•[IGFBP7] was predictive of AKI with an area under the receiver operating characteristic (ROC) curve as high as 0.9. Moreover, urinary [TIMP-2]•[IGFBP7] outperformed serum creatinine and urinary NGAL. In a subgroup analysis of the Sapphire and Topaz studies (conducted in Europe and the United States), urinary [TIMP-2]•[IGFBP7] predicted AKI with an AUC of 0.84 in patients who had cardiac surgery [19]. However, to our knowledge, urinary [TIMP-2]•[IGFBP7] has not been investigated in hospitals in Asia. In our study, we investigated the use of [TIMP-2]•[IGFBP7] for prediction of AKI in a cohort of adults undergoing cardiac surgery in China.

## Methods

### Study design and participants

We conducted a prospective observational study after obtaining approval from the institutional review board of the ethics committee of Zhongshan Hospital. Adult patients were screened for enrollment prior to cardiac surgery at Zhongshan Hospital in Shanghai, China, between May 01, 2016 to May 31, 2016. All enrolled patients provided written informed consent. Exclusion criteria were age less than 18 years, chronic dialysis, and prior renal transplantation. Urine samples for measurement of [TIMP-2]•[IGFBP7] were collected prior to surgery, in 2-h intervals from 0 h to 12 h after ICU admission, and 24 h after ICU admission to characterize the time-profile of [TIMP-2]•[IGFBP7]. Clinical data for the study were abstracted from hospital records and included patient demographics, medical history, surgical procedure, and serum creatinine and urine output data for AKI assessment.

### Endpoints

The primary endpoints were AKI and moderate-to-severe AKI within 7 days following surgery. AKI was defined according to the Kidney Disease: Improving Global Outcomes (KDIGO) criteria [10]. Moderate-to-severe AKI was defined as KDIGO stage 2 or 3 AKI. As in prior studies of cardiac surgery patients [15, 17, 18], both urine output and serum creatinine were used to determine AKI stages. Serum creatinine results from blood samples collected at hospital admission were used as the baseline values for AKI staging.

### Urine sample analysis

Urine samples were analyzed for TIMP-2 and IGFBP7 within 1 h of collection using the NephroCheck Test and Astute140 Meter (Astute Medical, San Diego, CA). The Astute140 Meter reports the product of the two protein concentrations ([TIMP-2]•[IGFBP7]) in units of (ng/mL)^2^/1000.

### Statistical analysis

To assess the predictive ability of [TIMP-2]•[IGFBP7] for the primary endpoints, empirical receiver operating characteristic (ROC) curves, area under the ROC curve (AUC), sensitivity, specificity, negative predictive value (NPV), and positive predictive value (PPV) were calculated. The 95% confidence intervals (CI) for the AUC and operating characteristics were determined using bootstrap sampling and the Clopper-Pearson method, respectively. To investigate the [TIMP-2]•[IGFBP7] time course, mean and standard error of the [TIMP-2]•[IGFBP7] values at each urine sample collection time were determined for endpoint positive and endpoint negative patients. Categorical variables were compared between groups using Fisher exact test. Continuous variables were compared using the Wilcoxon rank-sum test. Two-sided *P* -values <0.05 were considered statistically significant. Statistical analyses were performed using SAS 9.4 (SAS Institute, Cary, NC).

A clinical risk model for prediction of AKI was constructed starting from all clinical variables significantly (*P* < 0.05) associated with AKI (Table 1). Goodness of fit was assessed with the Hosmer-Lemeshow test. Category free net reclassification improvement (cfNRI), integrated discrimination improvement (IDI), and AUC difference were used to assess the improvement in predictive ability with the addition of [TIMP-2]•[IGFBP7] to the model.

**Table 1:** Baseline Characteristics for All patients and by AKI Status

	All	AKI	No AKI	*p* value
Subjects	57	20	37	
Male	41 (71.9%)	19 (95.0%)	22 (59.5%)	0.005
Age (Years)	60 (49–65)	64 (58–69)	53 (46–63)	0.01
Comorbidities				
Hypertension	22 (38.6%)	13 (65.0%)	9 (24.3%)	0.004
Diabetes mellitus	8 (14.0%)	3 (15.0%)	5 (13.5%)	>0.99
Cerebral infarction	3 (5.3%)	2 (10.0%)	1 (2.7%)	0.28
Hyperlipidemia	3 (5.3%)	3 (15.0%)	0(0%)	0.04
Chronic renal insufficiency	2 (3.5%)	2 (10.0%)	0(0%)	0.12
COPD/Emphysema	2 (3.5%)	1 (5.0%)	1 (2.7%)	>0.99
NYHA Class				0.26
I	5 (8.8%)	1 (5.0%)	4 (10.8%)	
II	24 (42.1%)	6 (30.0%)	18 (48.6%)	
III	27 (47.4%)	13 (65.0%)	14 (37.8%)	
IV	1 (1.8%)	0(0%)	1 (2.7%)	
EuroSCORE	3 (2–4)	4 (2–5)	3 (2–3)	0.09
Pre-operative serum creatinine (μmol/L)	78 (68–87)	85 (77–88)	75 (64–84)	0.02
Prior exposure to nephrotoxic drugs	5 (8.8%)	3 (15.0%)	2 (5.4%)	0.33
Prior exposure to radiocontrast agents	35 (61.4%)	15 (75.0%)	20 (54.1%)	0.16
Time from radiocontrast to surgery (d)	2 (1–5)	2 (1–3)	4 (2–6)	0.03
Procedures				
Mitral valve replacement	17 (29.8%)	5 (25.0%)	12 (32.4%)	0.76
Aortic valve replacement	17 (29.8%)	12 (60.0%)	5 (13.5%)	<0.001
Coronary artery bypass graft	12 (21.1%)	3 (15.0%)	9 (24.3%)	0.51
Valvuloplasty	17 (29.8%)	8 (40.0%)	9 (24.3%)	0.24
Cardiopulmonary bypass	42 (73.7%)	18 (90.0%)	24 (64.9%)	0.06
CPB time (minutes)	72 (63–108)	87 (66–139)	71 (58–93)	0.15
Aortic cross clamp time (minutes)	47 (35–66)	53 (30–92)	47 (35–63)	0.50
ICU Length of Stay (h)	23 (20–66)	34 (21–92)	22 (20–45)	0.14
Hospital Length of Stay (d)	12 (10–14)	12 (9–14)	12 (10–14)	0.51

*AKI* acute kidney injury, *COPD* chronic obstructive pulmonary disease, *NYHA* the New York Heart Association, *CPB* cardiopulmonary bypass

Qualitative variables are shown as N (%), quantitative variables as median with first and third quartiles (interquartile range)

## Results

### Patient characteristics

We enrolled 57 cardiac surgery patients. Twenty (35.1%) patients developed AKI within 7 days of surgery, and 6 (10.5%) patients developed stage 2–3 AKI. Two patients with stage 3 AKI received renal replacement therapy. Compared with patients who did not develop AKI, AKI patients were older, more likely to be male, and more likely to have a history of hypertension and hyperlipidemia (Table 1). AKI patients had higher preoperative serum creatinine levels, more recent exposure to radiocontrast agents, and were more likely to have undergone aortic valve replacement than non-AKI patients. Though not statistically significant (*P* = 0.06), cardiopulmonary bypass was more common among AKI patients than non-AKI patients.

### Biomarker performance

**Fig. 1:**
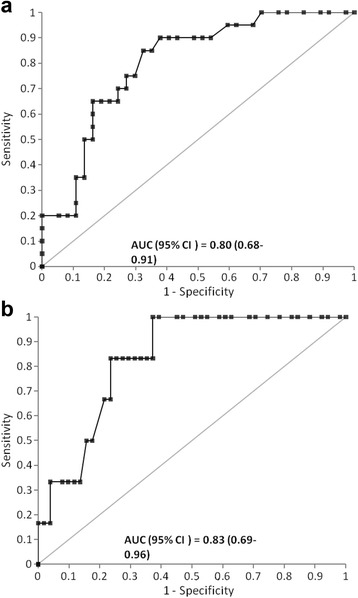
ROC curves for prediction of AKI using [TIMP-2]•[IGFBP7] results from urine samples collected 4 h after ICU admission. **a** prediction for all AKI, AUC = 0.80 (95% confidence interval 0.68 to 0.91, **b** prediction for stage 2–3 AKI, AUC = 0.83 (95% CI 0.69 to 0.96). *AKI* acute kidney injury, *AUC* the area under the ROC curve

The AUC (95% CI) of [TIMP-2]•[IGFBP7] four hours after ICU admission was 0.80 (0.68–0.91) for prediction of any AKI stage and 0.83 (0.69–0.96) for prediction of stage 2–3 AKI (Fig. 1). Table 2 showed operating characteristics for different [TIMP-2]•[IGFBP7] cutoffs, including the previously validated high sensitivity [TIMP-2]•[IGFBP7] cutoff of 0.3 and the high specificity cutoff of 2.0. The sensitivity was 75% for any AKI and 100% for stage 2–3 AKI at the 0.3 cutoff, and the specificity was 100% for any AKI and 96% for stage 2–3 AKI at the 2.0 cutoff. The NPV was 84% for any AKI and 100% for stage 2–3 AKI at the 0.3 cutoff, and the PPV was 100% for any AKI and 50% for stage 2–3 AKI at the 2.0 cutoff.

**Table 2:** [TIMP-2]•[IGFBP7] operating characteristics at 4 h after ICU admission for prediction of AKI

A. Endpoint is any AKI				
[TIMP-2]•[IGFBP7] Cutoff	Sensitivity	Specificity	NPV	PPV
0.3	0.75 (0.51–0.91)	0.70 (0.53–0.84)	0.84 (0.66–0.95)	0.58 (0.37–0.77)
0.5	0.50 (0.27–0.73)	0.84 (0.68–0.94)	0.76 (0.60–0.88)	0.63 (0.35–0.85)
1.0	0.20 (0.06–0.44)	0.95 (0.82–0.99)	0.69 (0.54–0.81)	0.67 (0.22–0.96)
2.0	0.20 (0.06–0.44)	1.00 (0.91–1.00)	0.70 (0.56–0.82)	1.00 (0.40–1.00)
B. Endpoint is stage 2–3 AKI				
0.3	1.00 (0.54–1.00)	0.61 (0.46–0.74)	1.00 (0.89–1.00)	0.23 (0.09–0.44)
0.5	0.67 (0.22–0.96)	0.76 (0.63–0.87)	0.95 (0.83–0.99)	0.25 (0.07–0.52)
1.0	0.33 (0.04–0.78)	0.92 (0.81–0.98)	0.92 (0.81–0.98)	0.33 (0.04–0.78)
2.0	0.33 (0.04–0.78)	0.96 (0.87–1.00)	0.92 (0.82–0.98)	0.50 (0.07–0.93)

*TIMP-2* urinary tissue inhibitor of metalloproteinase 2, *IGFBP7* insulin-like growth factor binding protein 7, *ICU* intensive care units, *AKI* acute kidney injury, *NPV* negative predictive value, *PPV* positive, predictive value

### Biomarker time course

**Fig. 2:**
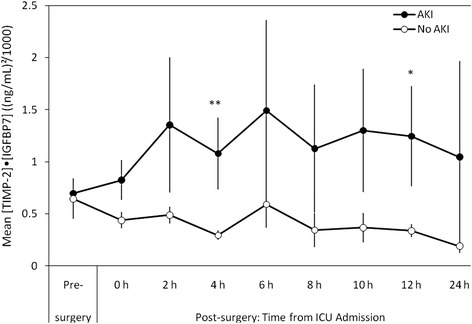
Mean [TIMP-2]•[IGFBP7] values in patients who developed AKI and those who did not. Times post-surgery are relative to ICU admission. Error *bars* show standard error. *p < 0.05, **p < 0.001

Mean (SE) [TIMP-2]•[IGFBP7] pre-surgery values were nearly the same in patients who did and did not develop AKI (0.70 (0.11) compared with 0.65(0.20), respectively). Following surgery, mean (SE) [TIMP-2]•[IGFBP7] values from patients who developed AKI increased to a peak of 1.49 (0.87) at 6 h after ICU admission; while mean (SE) [TIMP-2]•[IGFBP7] values from non-AKI patients decreased to a nadir of 0.19 (0.02) at 24 h after ICU admission (Fig. 2). At 4 h after ICU admission, mean (SE) [TIMP-2]•[IGFBP7] values were 1.08 (0.34) and 0.29 (0.05), respectively, for AKI and non-AKI patients. The difference in post-surgical [TIMP-2]•[IGFBP7] values between AKI and non-AKI patients was statistically significant at 4 h and 12 h after ICU admission.

### Clinical model

A clinical risk model for the prediction of AKI was constructed using backward selection. The selected variables were sex, hypertension and preoperative serum creatinine (Table 3). The AUC (95% CI) of the model increased from 0.83 (0.73–0.91) to 0.94 (0.83–0.98) with the addition of urinary [TIMP-2]•[IGFBP7] to the model (*P* = 0.03). The adjusted odds ratio (95% CI) for log_10_-transformed [TIMP-2]•[IGFBP7] was 115 (4–2156), *P* = 0.01. Reclassification analysis also showed an improvement in model performance with addition of [TIMP-2]•[IGFBP7] (cfNRI = 1.22 (95% CI 0.79 to 1.58), *P* < 0.001, and IDI = 0.19 (95% CI 0.07 to 0.31), *P* = 0.001).

**Table 3:** Adjusted odds ratios and AUC for prediction of AKI

	Clinical Model Alone		Clinical Model with [TIMP-2]•[IGFBP7]	
Adjusted Odds Ratio	Value (95% CI)	*P*	Value (95% CI)	*P*
Sex (Male/Female)	11.5 (1.6–107.4)	0.04	9.5 (2.6–83.6)	0.03
Hypertension	5.7 (1.7–18.0)	0.03	6.4 (3.2–35.7)	0.04
Preoperative creatinine >78 μmol/L	3.8(1.2–12.3)	0.01	4.0 (0.9–21.5)	0.04
[TIMP-2]•[IGFBP7]^a^	--	--	115 (4–2156)	0.01
Model AUC^b^	0.83(0.73–0.91)	<0.001	0.94 (0.83–0.98)	<0.001

*AUC* areas under the curves, *AKI* acute kidney injury, *TIMP-2* urinary tissue inhibitor of metalloproteinase 2, *IGFBP7* insulin-like growth factor binding protein 7

^a^Values were log10 transformed

^b^AUC for clinical model with [TIMP-2]•[IGFBP7] was significantly greater than the AUC for the clinical model alone, *p* = 0.03

## Discussion

Our study is the first known investigation of the performance of [TIMP-2]•[IGFBP7] in a Chinese population of patients undergoing cardiac surgery. The cell cycle arrest biomarkers TIMP-2 and IGFBP7 are released in the earliest stages of injury by renal tubule cells that have become stressed from kidney exposures that can lead to AKI [12, 20]. Elevated urinary [TIMP-2]•[IGFBP7] levels thus indicate renal tubule cell stress that precedes AKI, and this is believed to be the reason that [TIMP-2]•[IGFBP7] levels correspond to risk for AKI [12, 14, 20, 21]. Major surgery including cardiac surgery, complications such as hypotension or infection following surgery, and nephrotoxic drugs commonly prescribed in surgical patients are potential kidney exposures that can stress renal tubule cells and thus cause AKI [10], making cardiac surgery patients an appropriate at-risk population for risk stratification with the [TIMP-2]•[IGFBP7] test.

We chose to evaluate the [TIMP-2]•[IGFBP7] test 4 h after ICU admission for several reasons. First, this time is similar to that evaluated in prior studies of non-Asian cardiac surgery patients [15, 18]. Second, patients should be starting to stabilize at this time, and it is therefore a good time to evaluate which patients may be experiencing increased renal tubule cell stress, putting them at risk for developing a serious post-operative AKI complication. Such patients are good candidates to receive preventive measures that have been outlined in the KDIGO guideline [10] and elsewhere [20] for high-risk patients.

In our study, [TIMP-2]•[IGFBP7] measured in urine collected 4 h after ICU admission was predictive of both any stage of AKI and stage 2–3 AKI. The sensitivity at the previously validated 0.3 cutoff was 75% for any AKI and 100% for stage 2–3 AKI, and the specificity at the previously validated 2.0 cutoff was 100% for any AKI and 96% for stage 2–3 AKI.

Multiple studies in Germany have investigated urinary [TIMP-2]•[IGFBP7] for prediction of AKI following cardiac surgery in adults [15, 17, 18]. These studies either specifically enrolled patients undergoing coronary artery bypass graft (CABG) surgery or enrolled cardiac surgery patients deemed to be high risk for AKI as defined by a Cleveland Clinic Foundation Score [22] ≥ 6. Unlike in these studies, we did not restrict our enrolled cohort by type of cardiac surgery or pre-surgery risk. Yet, our results are strikingly similar to those of these other studies. In the CABG study, for which the endpoint was stage 2–3 AKI, the AUC (95% CI) of urinary [TIMP-2]•[IGFBP7] at 4 h after surgery was 0.86 (0.72–1.00) as compared to 0.83 (0.69–0.96) at 4 h after ICU admission for the same endpoint in our study.

Addition of urinary [TIMP-2]•[IGFBP7] to a clinical risk model significantly improved model performance, demonstrating that [TIMP-2]•[IGFBP7] provides critical information about AKI risk that is not obtainable from clinical risk factors alone. Our study also found that preoperative serum creatinine was significantly higher in AKI group and it is a risk factor for the prediction of AKI after cardiac surgery similar to previous studies in which subjects with higher serum creatinine developed acute or chronic kidney dysfunction after surgery [22, 23]. Of course the combination of creatinine and biomarkers is the future of AKI prediction. It is important to note that clinical risk prediction models derived from small data sets such as ours are “over-trained” and their performance thus overestimated. Therefore, although the AUCs for our clinical models (0.83 for clinical variables alone and 0.94 for clinical variables plus [TIMP-2]•[IGFBP7]) are high, clinicians should not rely on our clinical model for risk assessment. Nevertheless, the results are important because they show that [TIMP-2]•[IGFBP7] provides valuable information about AKI risk even when clinical risk factors are considered.

In our study, average urinary [TIMP-2]•[IGFBP7] levels appeared to be on the rise at the time of ICU admission and reached maximum levels 2–6 h after ICU admission in patients who developed AKI. In contrast, in patients who did not develop AKI, average urinary [TIMP-2]•[IGFBP7] levels appeared to slowly decrease after ICU admission. Most prior studies of cardiac surgery patients [15, 17, 18] have shown significant elevations of [TIMP-2]•[IGFBP7] 4 h after the end of cardiopulmonary bypass (CPB) in patients who developed AKI, similar to the measurement time in our study. However, two prior studies [17, 24] have shown maximum predictive ability the morning after surgery. Although it is usually assumed that the earliest injury in cardiac surgery associated AKI occurs during the surgery, it is possible in some patients that significant post-operative kidney exposures such as episodes of hypotension, developing infections or nephrotoxic drugs cause kidney stress and AKI to start developing during the post-operative period. This may explain why the maximum predictive ability of [TIMP-2]•[IGFBP7] occurred early after surgery in some studies (including ours) and later in other studies. This hypothesis requires additional investigation, but suggests that repeated testing might be appropriate in cases in which significant post-operative exposures might exist. Indeed, one study [15] found that use of three measurements (4, 12 and 24 h after CPB) had greater predictive value (AUC = 0.90) than a single measurement 4 h after CBP (AUC = 0.81).

Although all patients experiencing significant kidney exposures are at increased risk of AKI, only a small fraction (about 10% in our study) will develop the severest forms of AKI (stage 2–3). The [TIMP-2]•[IGFBP7] test provides a quantitative indication of risk (renal tubule cell stress) for each particular patient, with higher test values indicating higher stress and thus higher risk. Two well-established cutoffs, one at 0.3 that is highly sensitive and one at 2.0 that is highly specific provide risk stratification that allows the physician to apply preventative measures such as enhanced hemodynamic monitoring and modified drug dosing commensurate with the risk level [20]. Our study for the first time verifies these cutoffs in a Chinese population. Therefore, we can expect that protocols developed in other countries for using the test to help manage patients at risk for AKI will be relevant to patients undergoing cardiac surgery in China.

## Conclusions

The prediction performance of urine [TIMP-2]•[IGFBP7] in a Chinese cardiac surgery population is good for all of the AKI patients, not just stage 2–3 AKI. It is even better than that reported previously in non-Asian populations.

## Abbreviations

AKI: Acute kidney injury
AUC: The area under the ROC curve
CABG: Coronary artery bypass graft
cfNRI: Category free net reclassification improvement
CI: Confidence intervals
COPD: Chronic obstructive pulmonary disease
CPB: Cardiopulmonary bypass
ICU: Intensive care units
IDI: Integrated discrimination improvement
IGFBP7: Insulin-like growth factor binding protein
KDIGO: Kidney Disease: Improving Global Outcomes
NPV: Negative predictive value
NYHA: The New York Heart Association
PPV: Positive predictive value
ROC: Receiver operating characteristic curve
TIMP-2: Tissue inhibitor of metalloproteinase 2
